# In Search of Conceptual Clarity About the Structure of Psychopathic Traits in Children: A Network-Based Proposal

**DOI:** 10.1007/s10578-023-01649-z

**Published:** 2024-01-18

**Authors:** Laura López-Romero, Henrik Andershed, Estrella Romero, Matti Cervin

**Affiliations:** 1 Departamento de Psicología Clínica y Psicobiología, Facultad de Psicología, Universidade de Santiago de Compostela, Rua Xose María Suárez Núñez S/N, Campus Sur, 15782 Santiago de Compostela, Spain; 2 Örebro University, Örebro, Sweden; 3 Lund University, Lund, Sweden

**Keywords:** Psychopathic traits, Children, Network structure, Prediction, Conduct problems

## Abstract

Psychopathic traits in childhood have been revealed as potential identifiers of risk, being predictive of later forms of behavioral maladjustment. Yet, it is still under debate how psychopathic traits in children should be best conceptualized and which are the core dimensions for construct definition and prediction. The present study aims to examine the structure of psychopathic traits in childhood, and its predictive value, by using a combination of traditional factor analysis and more recent network-based methods. Data on psychopathic traits, as measured by the *Child Problematic Traits Inventory* (CPTI), were collected in a large sample of children (n = 2454; 48.2% girls), aged 3 to 6 at the onset of the study (*M*age = 4.26; SD = 0.91), who were followed-up one and two years later using parent- and teacher-reports. Results showed that psychopathic traits measured via CPTI are best conceptualized as five latent factors encompassing *grandiosity*, *deceitfulness*, *callousness*, *impulsivity* and *need of stimulation*, a result that converged across informants and time. Callousness and grandiosity emerged as central traits using network analysis of parent-reports, while deceitfulness was most central using teacher-reports. Finally, callousness, impulsivity and deceitfulness emerged as the best predictors of concurrent, prospective and stable conduct problems. These results provide a refined structure of psychopathic traits in children that better accounts for the core elements of the construct. Additional theoretical and practical implications will be discussed in terms of assessment, diagnostic classification and tailored prevention/intervention.

**Supplementary Information:**

The online version contains supplementary material available at 10.1007/s10578-023-01649-z.

## Introduction

Psychopathic personality traits have emerged as an important construct in understanding child conduct problems (CP) [1]. Over the past two decades, extensive evidence has been collected on their early identification (e.g., [2]), stability (e.g., [3]) and predictive value (e.g., [4]). The presence of psychopathic traits at early developmental stages has consistently been linked to problematic behaviors and negative outcomes, including more serious, persistent and aggressive patterns of child CP, and poorer or different response to treatment [4, 5].

Psychopathic personality has been commonly defined as a multidimensional construct encompassing a constellation of co-occurring interpersonal (e.g., grandiosity, deceitfulness, manipulation), affective (e.g., lack of empathy, callousness, shallow affect) and behavioral/lifestyle traits (e.g., impulsivity, sensation seeking, irresponsibility) [2, 6, 7]. Research conducted in childhood has mainly focused on the role of the affective dimension of the construct, namely Callous-unemotional (CU) traits, which theoretically encompasses traits within three subdimensions: Callousness, Uncaring and Unemotional [8]. Some consider CU traits to be the core dimension of the psychopathy construct in childhood and adolescence [9], and that such traits characterize an etiological and clinically distinctive subgroup of problematic children (see [5]), for a detailed review on this topic). As a result, CU traits have become increasingly recognized in theoretical models and empirical studies aiming to understand CP, and a CU-based specifier (i.e., “with limited prosocial emotions”, LPE) was added for the diagnosis of conduct disorder (CD) in DSM-5 [10] and ICD-11 [11].

While the CU-based conceptualization has resulted in great advances in the understanding of psychopathic traits in children, recent studies suggest that all three dimensions of the psychopathic construct may be important to predict more serious CP [4, 12]. In this regard, high levels of all three psychopathy dimensions have been shown to be more strongly related to child and youth CP, measured both concurrently and prospectively, than CU traits alone (e.g., [13–15]), even after controlling for other relevant risk factors (e.g., irritability, attention deficit hyperactivity symptoms) [16, 17]. Consequently, it has been argued that other psychopathic dimensions, and not only CU traits, should be considered in developmental and predictive models of CP and related negative outcomes (e.g., [4, 12, 18]), raising an active and constructive debate around which psychopathic dimensions are important for CP (e.g., [19, 20], see also [21, 22]).

### The Structure of Child Psychopathic Traits

Structures of psychopathological and behavioral symptoms are often examined using factor analysis, which helps organize patterns of covariance among specific symptoms. From a factor analytic approach, psychopathic traits in children have usually been structured under three (e.g., [2, 23, 24]) or four dimensions (e.g., [25]), including interpersonal, affective and behavioral/lifestyle traits. The *Child Problematic Traits Inventory* (CPTI; [2], constitutes one of the most comprehensive measures to assess psychopathic traits from early childhood and includes 28 items, broadly linked to the Grandiose-Deceitful (GD), Callous-Unemotional (CU) and Impulsive-Need of stimulation (INS) aspects of psychopathic traits. Previous validation studies have found that the 3-factor structure of the CPTI, using both the parent- and teacher-reported versions, can explain item covariance in an adequate way in different samples, contexts and settings (e.g., [24, 26–28]). Nevertheless, available research has left some room for improvement about how to best structure psychopathic traits in childhood. In this regard, psychopathic personality has usually been defined by broad dimensions (e.g., grandiose-deceitful) that can be narrowed into more refined traits (e.g., grandiosity, manipulation, deceitfulness). Further, research examining how the CPTI factors relate to each other has been scarce. This is an important gap in the literature as the internal structure of the CPTI may shed new light on how psychopathic traits are related in children and, even more interesting, how important each dimension is to overall define the construct and better predict CP.

A novel approach to understand relations among constructs, traits or items is *network analysis*, an analytical approach that has been increasingly applied to different forms of psychopathology, including depression and anxiety (e.g., [29]) or obsessive–compulsive disorder, which is known for its heterogeneity and overlapping yet still distinct symptom dimensions [30]. The network approach outlines how core elements of a construct are uniquely related. Specifically, by using network analytic techniques, unique associations among all included nodes in a construct are estimated [31]. Within network terminology, unique associations are referred to as edges and by using information from all edges in a network, the overall network structure can be graphically displayed. In such a graph, nodes with many edges to other nodes are placed centrally and nodes with a strong edge are placed closely. Symptoms of similar type or with a strong causal/reciprocal connection (e.g., lying and peer rejection) are expected to be well-connected and are likely to share connections to other symptoms in the network. Conceptually, this may resemble item loadings in a factor analysis, except the network approach does not relate the structural properties of the network back to a latent cause. Instead, the network approach is interested in the unique connections between nodes, such as bridge nodes that help to explain why two items that appear conceptually distinct can exist within the network of a single construct [31]. More specifically, rather than assuming that there is a latent construct of psychopathy, from the network approach one could assume that the interactions between the items would constitute the construct of psychopathy in itself [32]). In sum, network analysis can help to clarify how psychopathic traits are structured in childhood, disentangling whether some dimensions are more central than others, both within the structure and in relation to outcomes.

The application of network analysis to the structure of psychopathy has been relatively scarce, with most studies being conducted in adult populations using offending or forensic samples [32–34]. Results overall support that items within the affective dimension (e.g., callousness, lack of remorse and lack of empathy) are most central [33–35], that is, most densely connected within the overall psychopathy network. However, these results have not fully replicated across samples [34], with some studies also showing the importance of interpersonal and behavioral traits (e.g., [32]) even for later prediction [36].

To date, studies conducted with younger samples have mainly focused on identifying the most central symptoms/items of CU traits. In two samples of juvenile offenders and community youths, items from the Callousness dimension (e.g., lack of remorse and guilt, low empathy) were most central [37]. Similar results were observed in a high-risk sample of children and adolescents [38], with similar results across informants (i.e., parents and youths) and genders, and with items from Callousness acting as bridge nodes between CU traits and CP. To our knowledge, only one study has applied network analysis to examine the structure of CU traits in preschool children [39], showing that four items from the Callousness and Uncaring dimensions were most central. However, the small sample (*n* = 104) limits the generalization of results. In a recent study, Zhang et al. [40] examined the longitudinal network of psychopathic traits in a community sample of 248 Chinese children. Results showed that items assessing lack of remorse, not caring about other’s feeling and being deceitful, measured by the CPTI, were more central traits to the construct, with a network structure that remained relatively stable across a three-year period.

Prior research applying network analysis to psychopathic traits has analyzed networks of single items. Yet, the analyzed items have been drawn from scales developed to assess latent traits and thus often show considerable content overlap, which may result in strong edges between nodes (i.e., items). Further, the reliance on single items rests on the notion that each item is a perfect indicator of the construct it purposely is assumed to measure, which is unlikely in the realm of subjective reports. However, network analysis does not depend on item-level analyses. The techniques are equally applicable to broader dimensions or traits. Regarding psychopathic traits in children, a trait-level approach would resonate clearer with the current literature that is built around an understanding of dimensions and not single symptoms. Further, it would overcome the difficulties with item content overlap and unclear measurement error for individual items, making it possible to make more valid inference [41].

Based on the foregoing, the present study had three major aims. First, we aimed to identify the most valid dimensional structure of psychopathic traits in children. To succeed, we applied both factor and network analytic techniques to parent- and teacher reported CPTI data (item-level). Second, we aimed to identify the internal structure of the resulting CPTI dimensions. Here, we used network analysis on dimension-level data. Third, we aimed to explore which of the CPTI dimensions were most strongly associated with concurrent, prospective and stable CP. Our preliminary hypothesis suggests that traits within the interpersonal and affective dimension may play a central role in construct definition, across informants and across time, whilst all psychopathy dimensions would be predictive of later CP.

## Methods

### Participants

Data for the present study were collected in waves 1 to 3 of the *Estudio Longitudinal para una Infancia Saludable* (Longitudinal Study for a Healthy Childhood; [ELISA]), a prospective longitudinal study conducted in Galicia (NW Spain). Data collection started in 2017 (T1), encompassing preschool children who were born in 2011–2013, and with information provided by both parents and teachers. Only children with available data in some of the main study variables, namely psychopathic traits and conduct problems, were included in the present study (n = 2470). Sixteen participants with an affirmed diagnosis of, or being assessed for, autism spectrum disorder were excluded, resulting in a final sample of 2,454 children (48.2% girls), aged 3 to 6 (*M*_age_ = 4.26; *SD* = 0.91). A total of 72 public (79.2%), charter (18.1%), and private (2.8%) schools participated in the study, which were located in predominantly working-class communities, with low diversity in terms of ethnicity (93.9% of children were Spanish). Information was collected through 2250 parents’ reports (87.2% mothers), and 2407 reports from preschool teachers. Regarding children’s family background, 23.7% of mothers and 39.8% of fathers completed compulsory education, 47.4% and 31.2% completed higher education, and 28.9% and 29% completed vocational training studies.

Two follow-ups were conducted within one-year intervals. The first follow-up (T2) was conducted one year later in a sample of 2333 children (*M*_age_ = 5.35; *SD* = 0.92), with information provided by 1,993 parents (81.25% of the total sample) and 2170 teachers (88.46%). The level of attrition between T1-T2 participants was 4.69% considering the total sample, 11.42% based on parent-reports and 9.85% based on teacher-reports. The second follow-up (T3) was conducted two years following the initial assessment in a sample of 2272 children (*M*age = 6.33; *SD* = 0.92), with information provided by 1790 parents (72.98% of the total sample) and 2024 teachers (82.51%). The level of attrition between T1-T3 participants was 7.38% considering the total sample, 20.44% based on parent-reports and 15.91% based on teacher-reports. Comparisons between children with complete follow-up data (i.e., participation in three waves; n = 2.218; 90.4%), children who missed one of the follow-up studies (n = 172; 7%) and children with no follow-up data (i.e., participation only in T1; n = 63; 2.6%) revealed no significant differences in terms of gender, *χ*^*2*^ (2) = 4.92, *p* = 0.476; age *F* (2450) = 0.006, *p* = 0.994, and baseline levels of CP reported by parents, *F* (2213) = 0.763, *p* = 0.467. There were differences according to family’s SES, *F* (2235) = 13.03, *p* < 0.001, and the baseline levels of conduct problems reported by teachers, *F* (2409) = 4.42, *p* < 0.05, with lower levels of SES and higher levels of conduct problems for children who missed one of the follow-up studies.

### Measures

#### Psychopathic Traits

Both parents and teachers rated the 28 items of the CPTI [2] in all three waves of the study. Eight items intend to measure the interpersonal or Grandiose-deceitful (GD) psychopathy component (e.g., “Thinks that he or she is better than everyone on almost everything”), 10 items intend to measure the affective or Callous-unemotional (CU) psychopathy component (e.g., “Never seems to have bad conscience for things that he or she has done”), and 10 items intend to measure the behavioral or Impulsive-need of stimulation (INS) psychopathy component (e.g., “Provides himself or herself with different things very fast and eagerly”). The CPTI items were rated on the basis of how the child usually behaves rather than how he/she behaves at the moment, in a response scale ranging from 1 (*does not apply at all)* to 4 (*applies very well*). The optimal factor structure of CPTI was examined as part of the present study.

#### Conduct Problems

Both parents and teachers rated *The Conduct Problems Scale*, composed of 10 items (e.g., “Has been very angry”, and “Has beaten, torn, shoved, kicked, or thrown something on others without a reason”) that is closely based on DSM-IV (APA, 1994) criteria of oppositional defiant disorder (ODD) and CD, and were relevant to preschool children as well as older children and adolescents [2]. Items were scored using a 5-point response scale (1 = *never* to 5 = *very often*). Cronbach’s alpha (*α*) for the three waves ranged between 0.86 and 0.88 for parent reports, and between 0.93 and 0.94 for teacher reports. In line with prior work [17], children were classified as exhibiting *stable conduct problems* (CP) if they were 0.5 *SD* above the mean of the CP measure in T2 (4–6 years old) and T3 (5–7 years old).

### Procedure

The ELISA study was approved by the Bioethics Committee at the Universidade de Santiago de Compostela. A total of 126 public, charter and private schools were initially contacted in order to ask for potential collaboration. The initial contacts were made by phone, and information letters were subsequently sent by email. Once the school accepted the conditions and agreed to participate, families were contacted and invited to enrol in the study via information letters and group meetings in the schools, where a member of the research lab explained the conditions of the study. An active consent form was filled out by the families (approximately 25–50% response rate per school), after which the preschool teachers could also complete the questionnaires. Preschool teachers, who handed out the information to the parents, collected the informed consents. One teacher could complete the questionnaires for as many children in his/her classroom as there were written parental consent forms. Only one parent (i.e., mother, father, or principal caregiver) was asked to complete the questionnaires. Data collections took place during the Spring to assure that teachers have spent at least six months with the child before rating the questionnaire items. In all waves of the study, participants were given one month to complete the questionnaires. After that period, reminders were sent to those who were late, firstly by the preschool teacher and then directly by the ELISA staff via email. Neither families nor teachers received any monetary compensation for their participation in the study. Nonetheless, as a reward for their participation, all the schools received a set of educational games for preschoolers in T1, whilst both families and schools participated in a draw of several sets of books and educational games, valued between 50€ and 100€, at the end of the third wave data collection (T3).

### Statistical Analysis

#### Exploring the Optimal Factor Structure of the CPTI

To maximize the possibility to find the most adequate factor structure of the CPTI, two exploratory statistical frameworks were used. All exploratory models were based on parent-reported data from T1. First, we estimated the partial correlation network of the CPTI items using Copula gaussian graphical model estimation implemented in the R library *BGGM* (missing data were handled using multiple imputation with chained equations and predictive mean matching). We then inspected which items were strongly associated (i.e., correlated), with strong item-item associations being considered an indicator of a broader dimension. Zero-order polychoric correlations were used to estimate associations among items and we pooled strongly correlated items and reconducted the correlations until no correlations above 0.60 emerged. A correlation of 0.60 was selected because it indicates a moderate to strong correlation according to most criteria.

Second, we used exploratory factor analysis (EFA) to explore possible factor structures. EFA was based on the polychoric correlation matrix, and the Kaiser–Meyer–Olkin (KMO) test values were used to examine whether the items were suitable for EFA. KMO values indicate the proportion of variance in variables that might be explained by latent factors and values above 0.80 are considered to indicate that EFA is well suited. Bartlett’s test of sphericity was also used, where a significant test result (i.e., < 0.05) indicates that EFA is suitable. Horn’s parallel analysis was used to determine the number of factors to retain, and these factors were extracted using principal axis factoring and promax rotation.

#### Confirmatory Tests of Factor Models

The proposed model(s) identified using the methods described above, using parent-reported data from T1, were tested with new data (parent ratings from T2 and T3; and teacher-ratings from T1, T2 and T3) using confirmatory factor analysis (CFA). Model/data fit was evaluated using the Comparative Fit Index (CFI), Root Mean Square Error of Approximation (RMSEA), Standardized Mean Square Residual (SRMR), and Tucker-Lewis fit Index (TLI). Adequate model fit is indicated by higher CFI/TLI (values > 0.90 are indicative of adequate fit and values above 0.95 of good fit), and lower RMSEA and SRMR (values < 0.06 and 0.08, respectively, indicate good fit) [42]. Model fit of all models was contrasted with the fit of the original 3-factor CPTI model [2]. CFAs were run using the R library *lavaan* and because of the ordinal response scale, diagonally weighted least squares estimation and scaled fit indices were used and examined.

### Internal Structure of the CPTI Factors and Associations with Conduct Problems

When the best fitting factor model had been identified, we estimated the internal structure of the factors/dimensions by modeling them as a network. The R library *BGGM* and Copula gaussian graphical model estimation was used to identify edges among the dimensions. To control for false positive rate, we used 95% credible intervals (CIs) for the edges. All edges whose 95% CI did not include zero were considered statistically significant. The nodes and all significant edges were plotted as a network using the Fruchterman–Reingold algorithm implemented in the R-package *qgraph*. To examine whether any node was more strongly associated with other nodes in the network, we estimated the predictability (an *R*^*2*^-like measure) of each node. High predictability indicates that a node has many and strong edges with other nodes in the network. We compared the predictability of all nodes (i.e., factors/dimensions) and differences for which the 99% CI did not include zero were considered statistically significant. A 99% CI was used because of the large sample size and multiple comparisons.

To examine how the CPTI dimensions were associated with CP, we added a node to the network that indicated the degree of CP that the child exhibited at T1 (i.e., cross-sectional associations). To evaluate whether some CPTI dimension were more strongly related to CP than others, we compared all edges between the CPTI dimensions and CP. Differences for which the 99% CI did not include zero were considered statistically significant. Parent- and teacher-rated CPTI and CP data from T1 (ages 3–5) were used.

To examine which factors/dimensions were most important to predict later CP, we used regression models. CPTI dimensions were added as independent variables and later CP as the dependent variable. Two measures of later CP were used: (1) continuous parent- and teacher-rated CP scores at T3 and (2) stable CP defined as 0.5 *SD* above the mean of the CP measure at T2 and T3. We made inference based on the degree of explained variance of the full model and which independent variables were significantly associated with later CP. To make further inference, dominance analysis was used in which the unique contribution (in the form of explained variance) of each independent variable to later CP was estimated. For continuous CP scores, we used linear regression and for stable CP, we used logistic regression. For the logistic regression and the subsequent dominance analysis, Cox and Snell’s *R*^*2*^ were used to interpret explained variance. All predictive models were first conducted using only CPTI dimensions as independent variables and then by adding T1 CP as a covariate.

For comparative reasons, main analyses were replicated for the original 3-factor structure, with results presented as Supplemental material. All additional data and study materials are available upon request to the corresponding author.

## Results

### Exploring the Optimal Factor Structure of the CPTI Items

**Fig. 1:**
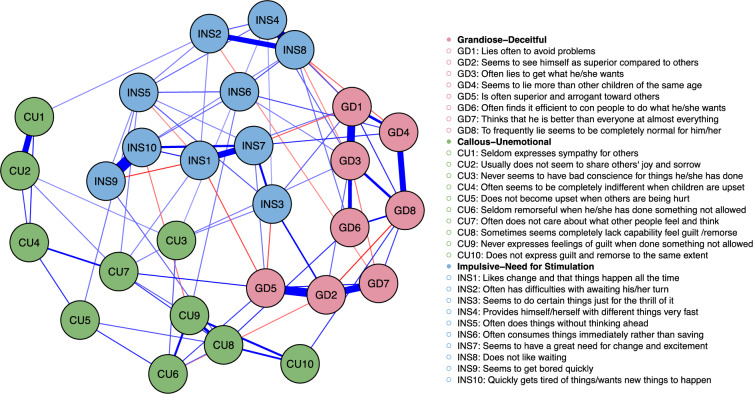
Partial Correlation Network of the CPTI Items. Each item is depicted as a circle and lines between circles indicate unique associations (i.e., a partial correlation for which the corresponding 95% credible interval does not include zero). Blue lines indicate a positive association and red lines a negative association. Items are grouped such that strongly associated items are placed closely (Color figure online)

Figure 1 displays the item network of CPTI based on parent-rated T1 CPTI data. Nodes are colored according to the original 3-factor structure. As can be seen in the figure, nodes formed fairly in line with their coloring. Several nodes were strongly associated even at the partial correlation level and in line with our analytical plan, we examined item correlations. Several node pairs correlated above 0.60 and 14 nodes were grouped into six variables. One more stage of pooling showed that no correlations above 0.60 were present. This led to a final solution with six factors in which nodes were grouped into factors pertaining information about (1) *grandiosity* (items: GD2, GD5, GD7), (2) *deceitfulness* (items: GD1, GD3, GD4, GD6, GD8), (3) *callousness* (items: CU4, CU5, CU6, CU7, CU8, CU9, CU10), (4) *impulsivity* (items: INS2, INS4, INS8) and (5) *need of stimulation* (items: INS9, INS10). A sixth factor that included items CU1 and CU2 also emerged (polychoric correlation = 0.63). Six items (INS1, INS3, CU3, INS5, INS6, INS7) did not correlate above 0.60 with any other item or any of the pooled item factors.

For the EFA, the mean KMO value was 0.92 and all items had a KMO value above 0.85 except INS1 (KMO value = 0.58), indicating that the INS1 item may not be a good indicator of a latent factor. Bartlett’s test was statistically significant (*p* < 0.001). In sum, the data were well suited for EFA except for item 1. Horn’s parallel analysis suggested seven factors with items INS3 and INS6 not loading above 0.50 onto any factor. The seven factors were extracted and explained 60% of the shared variance among items. The first five factors were identical to the five factors identified using the network analysis method with some minor exceptions. First, the EFA suggested that INS5 loaded onto the impulsivity factor (but the factor loading was below 0.50 and this was the lowest item loading for the factor). Second, the EFA suggested that CU1, CU2 and CU3 loaded onto the callousness factor, but these loadings were problematic because of double loadings onto other factors; further, these items had the weakest loadings among the items that loaded onto the callousness factor. EFA also proposed two additional two-item factors with one factor being indicated by INS1 and INS7, but these items only had a zero-order polychoric correlation of 0.44. The other two-item factor consisted of CU1 and CU2, but both items loaded more strongly onto the callousness factor.

By synthesizing results from both methods, we considered items INS1, CU1, CU2, INS3, CU3, INS5, INS6, INS7 to be diffuse/weak indicators of latent factors. To further examine the properties of these items, we selected to contrast, using CFA (see below), the narrower 5-factor model (*grandiosity, deceitfulness, callousness, impulsivity*, *need of stimulation*) and a broader six-factor model with identical factors but with items CU1, CU2 and CU3 being used as additional indicators of the callousness factor, and items INS1 and INS7 constituting an independent factor.

### Confirmatory Factor Analysis of Competing Models

CFA results for the two empirically derived models and for the original 3-factor model are presented in Table 1. The original 3-factor model showed adequate to poor model/data fit. Both the 5-factor and the 6-factor models showed good to excellent model/data fit using parent- and teacher ratings, but the 5-factor model showed better model/data fit for all indices for parent-ratings and for all but two indices for teacher-ratings. Because a different number of items was included in each model, we reconducted the CFAs by only comparing nested models (i.e., with identical items). This was done by omitting the eight diffuse/weak items described above. The six-factor model was not included as this model became identical to the 5-factor model when omitting diffuse/weak items. Results of the nested 3- and 5-factor models are at the bottom of Table 1. The 5-factor model showed clearly superior fit indices at all time-points, and this held true for both parent- and teacher-reported data. Fit indices for the 5-factor model were excellent at all time points and this model was deemed to show the most consistent data/model fit across both parent- and teacher-ratings.

**Table 1:** Fit indices of the original 3-factor model and the alternative 5-factor and 7-factor models

	CFI	TLI	RMSEA	SRMR
Non-nested models				
Parent-data T2, ages 4–6				
Original 3-factor model	0.931	0.925	0.068	0.066
Narrower 5-factor model	0.980	0.977	0.045	0.038
Narrower 6-factor model	0.969	0.964	0.050	0.044
Parent-data T3, ages 5–7				
Original 3-factor model	0.910	0.902	0.077	0.074
Narrower 5-factor model	0.966	0.960	0.060	0.046
Narrower 6-factor model	0.953	0.946	0.060	0.051
Teacher-data T1, ages 3–5				
Original 3-factor model	0.956	0.952	0.077	0.073
Narrower 5-factor model	0.985	0.982	0.061	0.037
Narrower 6-factor model	0.978	0.975	0.061	0.042
Teacher-data T2, ages 4–6				
Original 3-factor model	0.966	0.963	0.081	0.073
Narrower 5-factor model	0.985	0.982	0.061	0.037
Narrower 6-factor model	0.983	0.980	0.065	0.041
Teacher-data T3, ages 5–7				
Original 3-factor model	0.945	0.940	0.111	0.086
Narrower 5-factor model	0.981	0.978	0.087	0.045
Narrower 6-factor model	0.977	0.973	0.081	0.047
Nested models				
Parent-data T2, ages 4–6				
3-factor model	0.938	0.930	0.078	0.071
5-factor model	0.980	0.977	0.045	0.038
Parent-data T3, ages 5–7				
3-factor model	0.927	0.917	0.084	0.074
5-factor model	0.966	0.960	0.060	0.046
Teacher-data T1, ages 3–5				
3-factor model	0.969	0.965	0.085	0.077
5-factor model	0.985	0.982	0.061	0.037
Teacher-data T2, ages 4–6				
3-factor model	0.973	0.969	0.096	0.085
5-factor model	0.986	0.984	0.069	0.038
Teacher-data T3, ages 5–7				
3-factor model	0.948	0.941	0.142	0.105
5-factor model	0.981	0.978	0.087	0.045

T1 = Wave 1; T2 = Wave 2; T3 = Wave 3

The internal consistency of the items of each factor in the 5-factor model was good to excellent across parent- and teacher ratings (Cronbach’s alpha for parent ratings at T2 and T3: 0.82 to 0.93; alpha for teacher ratings at T2 and T3: 0.86 to 0.98). In the Supplementary Material (Table S1), we present the items of each factor in the 5-factor model and their standardized CFA factor loadings across parent- and teacher ratings at T2 and T3.

### Internal Structure of the CPTI Dimensions

**Fig. 2:**
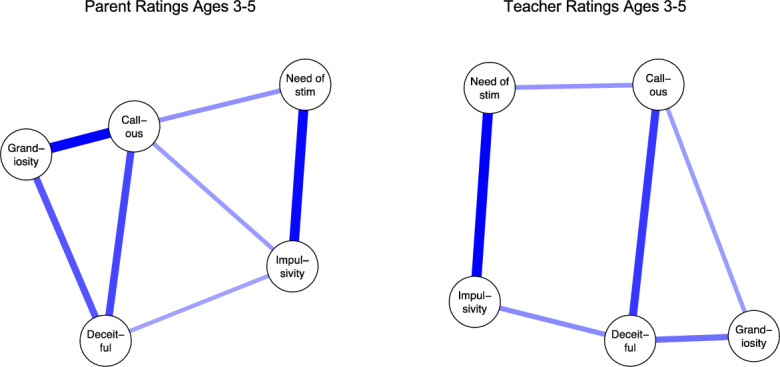
Internal Structure of the 5-factor CPTI Model. Each variable is depicted as a circle and lines between circles indicate a partial correlation for which the corresponding 95% credible interval does not include zero. Blue lines indicate a positive association. Variables are placed such that strongly associated variables are placed closely (Color figure online)

Zero-order Pearson correlations among the five new CPTI dimensions using parent-rated T1 data were all in the moderate range with the smallest correlation emerging between *impulsivity* and *grandiosity* (*r* = 0.27, *p* < 0.001) and the largest between *callousness* and *grandiosity* (*r* = 0.45, *p* < 0.001). Similar results emerged for teacher-ratings, but the correlations were overall larger, with the smallest being between *need of stimulation* and *grandiosity* (*r* = 0.35, *p* < 0.001) and the largest between *need of stimulation* and *impulsivity* (*r* = 0.67, *p* < 0.001). The network structure of the refined 5-factor model based on parent- and teacher reported data at T1 is in Fig. 2. In the parent-reported network, *callousness* had unique associations with all other dimensions. In the teacher-rated network, no dimension had unique associated with all other dimensions, but *callousness* and *deceitfulness* were each associated with three other dimensions. In both the parent- and teacher reported network, *callousness, deceitfulness* and *grandiosity* formed a community of variables that were closely associated alongside another community that included *impulsivity* and *need of stimulation*. In both the parent- and teacher-rated original 3-factor network, all variables were uniquely associated with each other (see Supplementary Material).

In the parent-rated 5-factor network, *grandiosity* (predictability: 41.5% [95%CI 37.9%–44.9%]) and *callousness* (predictability: 38.2% [34.9%–41.5%]) had significantly higher predictability (i.e., were more central) than the other dimensions: *deceitfulness* (predictability: 30.6% [27.1%–34.0%]), *need of stimulation* (predictability: 25.6% [22.2%–28.9%]), and *impulsivity* (predictability: 16.4% [14.3%–18.6%]). *Impulsivity* was less central than all other dimensions.

In the teacher-rated network, *deceitfulness* (predictability: 61.9% [58.9%–64.9%]) was more central than all other dimensions: *callousness* (predictability: 55.6% [52.5%–58.8%]), *need of stimulation* (predictability: 50.1% [47.7%–54.2]), *grandiosity* (predictability: 49.8% [46.7%–52.9%]), *impulsivity* (predictability: 44.0% [40.9%–47.1%]). Further, *impulsivity* was less central than all other dimensions. See Supplementary for predictability results for the original 3-factor CPTI structure.

### Cross-sectional Associations with CP

**Fig. 3:**
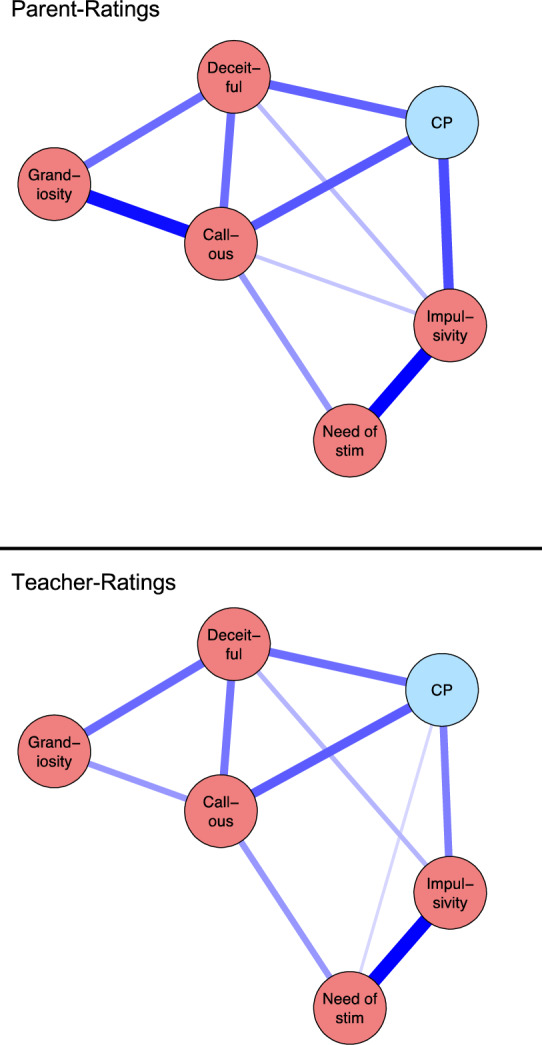
Associations between the Refined 5-factor Structure of the CPTI and Conduct Problems at wave 1. Each variable is depicted as a circle and lines between circles indicate unique associations (i.e., a partial correlations for which the corresponding 95% credible interval does not include zero). Blue lines indicate a positive association and red lines a negative association. Variables are placed such that strongly associated variables are placed closely (Color figure online)

The network of CPTI factors and CP, using data from T1, for the refined 5-factor model is presented in Fig. 3. For parent-ratings, *callousness* (edge to CP: 0.23), *deceitfulness* (edge to CP: 0.21) and *impulsivity* (edge to CP: 0.24) were uniquely associated with CP and were statistically significantly more strongly associated with CP than *grandiosity* (edge to CP: 0.07) and *need of stimulation* (edge to CP: 0.04). For teacher-ratings, *impulsivity* (edge to CP: 0.27), *callousness* (edge to CP: 0.34) and *deceitfulness* (edge to CP: 0.30) were significantly more strongly associated with CP than *need of stimulation* (edge to CP: 0.09) and *grandiosity* (edge to CP: 0.03) but not significantly different from each other.

### Predicting Later CP

A linear regression model that used the parent-rated continuous CP measure at T3 as the dependent variable and the five parent-rated CPTI dimensions at T1 as independent variables was statistically significant (*p* < 0.001) and explained 20.0% of the variance in later CP. All CPTI dimensions were significant predictors, *grandiosity* (β = 0.05, *p* = 0.04), *deceitfulness* (β = 0.11, *p* < 0.001), *callousness* (β = 0.21, *p* < 0.001), *impulsivity* (β = 0.18, *p* < 0.001), and *need of stimulation* (β = 0.08, *p* < 0.01). Dominance analysis showed that *callousness* explained most variance in later CP (6.5%) followed by *impulsivity* (5.4%), *deceitfulness* (3.7%), *need of stimulation* (2.4%) and *grandiosity* (2.1%).

An identical model but based on teacher-ratings was also significant (p < 0.001) and explained 23.2% of the variance in teacher-rated T3 CP scores. All CPTI dimensions except need of stimulation were significant predictors: *grandiosity* (β = − 0.07, *p* < 0.01), *deceitfulness* (β = 0.13, *p* < 0.001), *callousness* (β = 0.25, *p* < 0.001), and *impulsivity* (β = 0.20, *p* < 0.001). Dominance analysis showed that *callousness* explained most variance in later CP (7.7%) followed by *impulsivity* (6.0%), *deceitfulness* (5.5%), *need of stimulation* (3.1%) and *grandiosity* (0.9%, negative association in the regression model).

When we accounted for CP levels at T1, the model based on parent ratings was significant (*p* < 0.001) and explained 47.5% of the variation in later CP. The only CPTI dimensions that significantly predicted T3 CP were *need of stimulation* (β = 0.05, *p* = 0.03) and *callousness* (β = 0.05, *p* = 0.03); T1 CP was a strong predictor (β = 0.65, *p* < 0.001). Dominance analysis showed that T1 CP explained most variance (17.4%) followed by *callousness* (5.1%), *impulsivity* (4.1%), *deceitfulness* (3.8%), *need of stimulation* (2.3%) and *grandiosity* (1.0%). For teacher-ratings, the model was significant (*p* < 0.001) and explained 33.6% of the variation in later CP. Only *grandiosity* (β = − 0.08, *p* < 0.001) and *callousness* (β = 0.07, *p* = 0.01) were significant predictors among the CPTI dimensions and the association was negative for *grandiosity*. T1 CP was a strong predictor also for teacher-ratings (β = 0.55, *p* < 0.001) and dominance analysis showed that T1 CP explained most variance (19.8%) followed by *callousness* (4.4%), *deceitfulness* (3.4%), *impulsivity* (2.2%), *need of stimulation* (2.0%) and *grandiosity* (1.9%).

Last, we predicted stable CP. Using parent-ratings, the logistic regression that included only CPTI dimensions was significant and explained 18.0% of the variance (Cox and Snell’s pseudo R^2^). All CPTI dimensions were significant predictors of stable CP, *grandiosity* (standardized OR = 1.15, *p* = 0.05), *deceitfulness* (standardized OR = 1.28, *p* < 0.001), *callousness* (standardized OR = 1.52 *p* < 0.001), *impulsivity* (standardized OR = 1.33, *p* < 0.001), and *need of stimulation* (standardized OR = 1.21, *p* = 0.01). Dominance analysis showed that *callousness* explained most variance in stable CP (5.9%) followed by *deceitfulness* (4.2%), *impulsivity* (2.9%), *need of stimulation* (2.5%) and *grandiosity* (2.4%). The model for teacher-ratings was also significant and explained 22.1% of the variance (Cox and Snell’s pseudo R^2^) and all CPTI dimensions except *need of stimulation* were significant predictors, *grandiosity* (adjusted OR = 0.70 [negative association], *p* < 0.01), *deceitfulness* (adjusted OR = 1.37, *p* < 0.001), *callousness* (adjusted OR = 1.57, *p* < 0.001), and *impulsivity* (adjusted OR = 1.93, *p* < 0.001). Dominance analysis showed that *callousness* explained most variance in stable CP (7.1%) followed by *impulsivity* (5.8%), *deceitfulness* (4.1%), *need of stimulation* (3.2%) and *grandiosity* (2.0%).

When accounting for T1 CP, the model based on parent-ratings explained 33.6% of the variation (Cox and Snell’s pseudo R^2^) and only T1 CP was a significant predictor (standardized OR = 5.41, *p* < 0.001). Dominance analysis showed that T1 CP explained most variance (19.8%) followed by *callousness* (4.4%), *deceitfulness* (3.4%), *impulsivity* (2.2%), *need of stimulation* (2.0%) and *grandiosity* (1.9%). For teacher-ratings, the model explained 27.3% of the variation in stable CP and *impulsivity* (adjusted OR = 1.38, *p* < 0.01), *grandiosity* (adjusted OR = 0.75 [negative association], *p* < 0.01), and T1 CP (adjusted OR = 3.47, *p* < 0.01) were significant predictors. Dominance analysis showed that T1 CP explained most variance (10.0%) followed by *callousness* (5.6%), *impulsivity* (4.2%), *deceitfulness* (3.1%), *need of stimulation* (2.5%) and *grandiosity* (2.0%).

## Discussion

The present study intended to examine the core structure of psychopathic traits in early childhood and how empirically supported traits were associated with each other and concurrent and future CP. Using the 28 items included in the CPTI [2], we found that a refined 5-factor structure replicated across informants and time. This structure broadly includes the same traits as in the original 3-factor model but depicts a more fine-grained solution. Importantly, the five narrower dimensions were only moderately correlated, indicating that they capture partly unique information about psychopathic traits in children. When modeled as a network, findings showed that traits within the GD dimension (i.e., grandiosity and deceitfulness) and traits within the INS dimension (impulsivity and need of stimulation), can be considered partly independent features of psychopathic personality in childhood. Although moderately correlated, these narrower elements emerged as distinctive constructs that may differently contribute to the definition of psychopathic personality in children. In contrast, the CU dimension was refined by removing 3 items that reflected how children resonate with others’ feelings (e.g., “Usually does not seem to share others’ joy and sorrow”) but the other core features were retained in a single dimension. These results converge with previous studies, suggesting that callousness, and to some extent uncaring traits, might be the core features of the CU dimension in youths (e.g., [37–39]).

When examining the internal structure of the refined CPTI dimensions, we identified two community of features that clustered together, one encompassing grandiosity, deceitfulness and callousness, and the other encompassing impulsivity and need of stimulation. Importantly, this result replicated across informants. This structure clearly resembles the traditional definition of psychopathy, described as a constellation of co-occurring traits organized under two broad factors of affective-interpersonal (Factor 1), and behavioral-lifestyle traits (Factor 2) [43], suggesting a higher-order structure that has also been identified in childhood (e.g., [44]).

Using centrality estimates for each node within the networks, both interpersonal (i.e., deceitfulness and grandiosity) and affective traits (i.e., callousness) emerged as potential core elements of the psychopathy construct, a result that also converged between informants, and that is in line with previous network studies using the multidimensional construct of psychopathy in children [40] adult samples (e.g., [33, 35]). This is an important result since it provides additional support to consider traits within the CU dimension as central features of the construct, but not as the unique core dimension. The study of psychopathic personality at early developmental stages has been built-up from the assumption that CU traits represent the hallmark of the construct [9], being sometimes equated with psychopathic personality. However, CU traits only capture one psychopathy dimension, which has indeed been proved to identify a specific group of problematic children [5], but has sometimes failed to uniquely identify a higher risk profile [27]. Most of previous studies did not account for the potential co-occurrence with other psychopathic features, restraining the possibility to check whether other dimensions are also relevant for prediction [20]. In this regard, it has been observed that interpersonal features of the psychopathy construct have unique predictive value for certain negative outcomes, being as relevant as CU traits in designating a group of children with a specific pattern of behavioral and psychosocial maladjustment [12, 15, 19, 45]. Research on interpersonal callousness, a broad domain that accounts for both interpersonal and affective features of psychopathy, also supported its usefulness in childhood and adolescence [46, 47], being predictive of later forms of antisocial behavior and adult psychopathy (e.g., [48, 49]), and supporting the importance of both interpersonal and affective traits in designating a high-risk group of problematic youths.

### Reinforcing the Predictive Value of Child Psychopathic Traits

Current results also support a long-standing research line that consistently linked early psychopathic traits with concurrent, prospective and stable CP, even when controlling for concurrent CP (see [4, 5], for compelling reviews). More specifically, results raised callousness, impulsivity and deceitfulness as the strongest and clearest predictors of CP, measured concurrently, two-years later, and using a stable measure of high CP. Hence, these three specific dimensions play a central role not only in construct definition, but also in the prediction of more serious and persistent CP. Interestingly, these results overall held across informants, although in teachers’ reports, the influence of deceitfulness is not as strong as for callousness and impulsivity. Yet, all three dimensions showed closer associations with CP than grandiosity and need of stimulation. Of note, teacher-reported grandiosity showed a pattern of negative associations with both concurrent and longitudinal CP. This result contrasts with those obtained for the 3-factor model, in which the broad interpersonal dimension, comprised by grandiose-deceitful traits, showed a positive association with CP at three levels of measurement (concurrent, prospective and stable). How psychopathic personality is characterized may impact the extent to which it is predictive of negative outcomes [50]. By clearly depicting the construct of psychopathic personality in childhood, we identified a more refined picture of the centrality and predictive value of each specific trait. As a matter of fact, by splitting the INS domain into impulsivity and need for stimulation, we showed that impulsivity was the trait that was uniquely associated with CP, although it was the less central to the construct. Similarly, by splitting the GD domain into two more clearly defined dimensions, we showed that the link to CP was particularly carried by deceitfulness, whilst grandiosity was not a good predictor of later CP. In fact, as was previously mentioned, grandiosity was negatively linked to CP when teacher-reports were examined. It might be that teacher-appraised grandiosity capture aspects of this trait linked to beneficial aspects of functioning (e.g., self-efficacy, self-confidence, self-pride). These results would be in line with previous studies examining the potential adaptive value of grandiose narcissism or adaptive narcissism, which have been positively related to better psychological functioning in adulthood (e.g., [51]), and adolescence (e.g., [52]).

### Implications

A clearer conceptualization of the psychopathic construct early in development is key to provide evidence-based guidance at both theoretical and practical levels. Although much more research is needed, results from the current study provide support to address additional domains within psychopathic personality (e.g., interpersonal features; [45]), that have proved their relevance in predicting later behavioral and psychosocial problems when studying psychopathic traits in childhood and adolescence (e.g., [15, 19]). In this regard, the present study suggests that, in addition to callousness, deceitful and impulsivity traits may be important contributors to the development of CP during childhood.

Yet, it is important to further elucidate how distinctive psychopathic dimensions contribute to the overall construct and to CP. For instance, it has been suggested that traits within the behavioral dimension (i.e., impulsivity and need of stimulation), would better reflect ADHD behaviors, a well-established predictor of CP in childhood [19]. This rationale is based on correlational studies showing a strong association between INS traits and ADHD (e.g., [53]), whilst some others, even within the CPTI research, showed moderate levels of association (e.g., [24]). The hypothesis has also been specifically tested in a recent study that showed a substantial overlap between INS traits (presented with high levels of CP) and ADHD [54]. However, when multiple dimensions were used for subtyping purposes, the overlap with ADHD were not exclusive for INS traits, and it could be partially explained by concurrent CP. Hence, it is important to keep examining psychopathic traits, including INS traits, within the theoretical framework of psychopathy, as they might be adding some value beyond ADHD symptoms, particularly if they are presented in combination with interpersonal and affective traits [20]. Disentangling the core features that best contribute to define the psychopathy construct, and how they relate with CP, will provide additional support to further advance this endeavor.

By assuming the multidimensionality of the construct, with refined central features that can be identifiable and reliable assessed early in development [2], new advances can be delineated on how problematic traits can be configured into distinctive profiles, with distinctive traits permutations, and how these profiles are predictive of later CP. Similarly, the study of differential etiological mechanisms can also be addressed leading to elucidate whether previous findings on CU traits can be extrapolated to other psychopathy dimensions (e.g., [55]) or, in turn, whether the combination of high interpersonal, affective, and behavioral traits may identify a distinctive etiological subgroup of children at increased risk for later CP and related outcomes. These results will shed additional light on how psychopathic personality develops over time, how it should be integrated in developmental models and subtyping approaches of CP, how it relates with other forms of psychopathology and dysfunction, and, even more important, how we can work to prevent and potentially restrain the development of the most serious patterns of problematic behaviors.

We want to clarify that current results do not aim to question or invalidate the original three-factor model of the CPTI, which has been consistently replicated across samples, contexts, and languages (e.g., [2, 24, 26, 28]). Yet, based on the knowledge previously accumulated within the CPTI research, we aimed to move a step forward, and disentangle a more refined structure that may help to better account for the core features of the psychopathic personality construct in childhood. The better we know the construct, the more we will improve our predictive and developmental models of child CP. Further, more tailored preventive and intervention programs could be delineated based on specific traits that have proved to be central to the construct as well as for prediction of later CP. Importantly, before deriving practical applications, it is imperative to build a solid base of knowledge around how psychopathic traits are structured, how they related to each other, particularly across time, and how these relations are linked to different forms of behavioral, emotional and psychosocial maladjustment. Based on the previous experience with CU traits, currently included in diagnostic classification systems, new avenues to better inform how CP emerge and develop across childhood are guaranteed.

### Strengths, Limitations and Avenues for Future Research

Notwithstanding the strengths of the current study, which include the longitudinal design, with a large sample size, a multi-informant perspective, and the inclusion of network techniques to disentangle the core structure of psychopathic traits in childhood, some limitations merit mention. First, current results about a refined CPTI structure should be considered preliminary and need to be replicated, particularly in at-risk or clinic-referred samples, where higher levels of both psychopathic traits and CP are expected, and from cross-national samples. Second, psychopathic traits were only analyzed in relation to CP, but additional studies, covering other outcomes (e.g., ODD, CD, aggressive behavior) are needed. Third, potential gender differences should be addressed in future research. Although previous studies with the CPTI and other multidimensional measures (e.g., the Proposed Specifiers for Conduct Disorder; [56] showed structure invariance across preschool (e.g., [24, 25]) and school-aged boys and girls [57], some others have revealed that the structure of psychopathy might not be equal across gender groups, particularly in adult samples (e.g., [58]). Fourth, despite the prospective design, only a two-year period was covered. Studies spanning longer research intervals will allow to examine how the core structure of psychopathic personality remains stable across different developmental periods, as well as to elucidate the potential developmental relationship across psychopathy dimensions [19]. Finally, even though network analysis has been widely used to clarify the dynamic causal structure of mental disorders, this analytic approach is not exempt of criticism and limitations that have been the object of recent debates [59, 60]. Therefore, caution when interpreting these results should be encouraged, at least until new replication studies, preferably from multi-method approaches, provide additional support for this refined structure of psychopathic traits in childhood.

## Summary

Due to the importance of psychopathic traits to predict more serious and persistent patterns of child CP, it is crucial to further understand how psychopathic traits are structured in childhood, and which dimensions are central for construct definition and prediction. Current results, obtained in a large sample of preschool children, suggested a refined structure of psychopathic traits, with five thematically clear dimensions that were replicated across informants and ages. Results supported the importance of callousness and grandiosity for construct definition using parent-reports, while deceitfulness was most central using teacher-report. However, both interpersonal (i.e., deceitfulness) and behavioral traits (i.e., impulsivity) were central to the construct using both parent- and teacher-reports. Important results linking core psychopathy traits to CP also emerged, with callousness, impulsivity and deceitfulness being most clearly associated with concurrent and prospective CP. Overall, these are promising results that may help to derive a more refined conceptualization of the psychopathy construct in childhood, which may have important implications for construct definition, diagnostic classification and the development of more tailored prevention and intervention strategies.

## Supplementary Information

Below is the link to the electronic supplementary material.

Supplementary file1 (DOCX 259 KB)

## Data Availability

All data sets, scripts and materials are available upon request to the corresponding author.
