# Supplementary material for: In Search of Conceptual Clarity About the Structure of Psychopathic Traits in Children: A Network-Based Proposal
Source: Child Psychiatry Hum Dev. 2024 Jan 18;56(6):1–14. doi: 10.1007/s10578-023-01649-z (PMC12628477; doi:10.1007/s10578-023-01649-z)
Supplement: Supplementary file 1 — Supplementary file1 (DOCX 259 KB) [file 10578_2023_1649_MOESM1_ESM.docx]

**Supplementary material**

*for*

In Search of Conceptual Clarity about the Structure of Psychopathic Traits in Children:

A Network-based Proposal

**Table S1.** Items included in the refined 5-factor CPTI model and standardized factor loadings using confirmatory factor analysis with parent- and teacher-reported data.

|  |  | Factor loading  Parent-ratings  T2 | Factor loading  Parent-ratings  T3 | Factor loading  Teacher-ratings  T2 | Factor loading  Teacher-ratings  T3 |
| --- | --- | --- | --- | --- | --- |
| **Grandiosity** | |  |  |  |  |
|  | *Item 7. Seems to see himself as superior compared to others* | .85 | .85 | .95 | .92 |
|  | *Item 18. Is often superior and arrogant toward others* | .90 | .93 | .97 | .96 |
|  | *Item 24. Thinks that he is better than everyone at almost everything* | .81 | .79 | .96 | .95 |
| **Deceitfulness** | |  |  |  |  |
|  | *Item 5.* *Lies often to avoid problems* | .79 | .83 | .91 | .92 |
|  | *Item 9. Often lies to get what he/she wants* | .86 | .91 | .95 | .96 |
|  | *Item 15. Seems to lie more than other children of the same age* | .82 | .86 | .94 | .94 |
|  | *Item 21. Lies to get people to do what he/she wants* | .83 | .85 | .91 | .94 |
|  | *Item 26. To frequently lie seems to be completely normal for him/her* | .89 | .90 | .95 | .95 |
| **Callousness** | |  |  |  |  |
|  | *Item 11. Often seems to be completely indifferent when children are upset* | .75 | .79 | .90 | .86 |
|  | *Item 13. Does not become upset when others are being hurt* | .79 | .79 | .93 | .89 |
|  | *Item 17. Seldom remorseful when he/she has done something not allowed* | .81 | .83 | .95 | .92 |
|  | *Item 20. Often does not care about what other people feel and think* | .79 | .84 | .93 | .82 |
|  | *Item 22.* *Sometimes seems completely lack capability feel guilt /remorse* | .88 | .85 | .96 | .91 |
|  | *Item 25.* *Never expresses feelings of guilt when done something not allowed* | .83 | .78 | .94 | .91 |
|  | *Item 27.* *Does not express guilt and remorse to the same extent* | .85 | .86 | .95 | .91 |
| **Impulsivity** | |  |  |  |  |
|  | *Item 3.* *Often has difficulties with awaiting his/her turn* | .68 | .74 | .92 | .88 |
|  | *Item 10.* *Provides himself/herself with different things very fast* | .82 | .88 | .97 | .89 |
|  | *Item 19.* *Does not like waiting* | .83 | .84 | .94 | .91 |
| **Need of stimulation** | |  |  |  |  |
|  | *Item 23.* *Seems to get bored quickly* | .81 | .82 | .89 | .88 |
|  | *Item 28.* *Quickly gets tired of things/wants new things to happen* | .86 | .87 | .91 | .87 |

*Notes.* CPTI = The Child Problematic Traits Inventory.

**Predictability for original, 3-factor structure**

In the parent-rated original, three-factor network, *grandiose-deceitful* (predictability: 40.9% [38.0%-43.8%]) was more central than the two other variables, and c*allous-unemotional* (predictability: 36.2% [33.2%-39.1%]) was more central than *impulsive-need for stimulation* (predictability: 17.8% [15.7%-19.9%]). In the teacher-rated three-factor network, *grandiose-deceitful* (predictability: 61.2% [58.2%-64.3%]) and c*allous-unemotional* (predictability: 59.5% [56.5%-62.6%]) was more central than *impulsive-need for stimulation* (predictability: 14.3% [12.6%-16.1%]) but not statistically significantly different from each other.

**Associations between original, 3-factor structure and conduct problems**

Associations between CPTI factors and CP using the three-factor CPTI model are presented in **SFigure 1**. For parent-rated cross-sectional data, all three CPTI factors were significantly associated with CP (explained variance in CP: 31.3%) but *impulsive-need for stimulation* was significantly more strongly associated with CP than *grandiose-deceitful* and *callous-unemotional*; the two latter factors did not significantly differ in their association with CP. When the three CPTI factors were used to predict CP two years later (explained variance in CP: 19.9%), all three factors significantly predicted CP with no significant differences in the strength of these predictive associations. When predicting stable CP (explained variance in CP: 18.6%), again no significant differences emerged.

Cross-sectional teacher-rated data showed that all three factors were significantly associated with CP (explained variance in CP: 62.1%) and that *impulsive-need for stimulation* was significantly more strongly associated with CP than the other two factors. Further, *callous-unemotional* was significantly more strongly associated with CP than *grandiose-deceitful*. When teacher data was used to predict CP two years later, all three variables predicted CP (explained variance in CP: 26.9%) with no significant differences in the strength of these predictive associations. When predicting stable CP (explained variance in CP: 29.4%), *grandiose-deceitful* was a weaker predictor than the other two factors that did not differ.

**Figure S1.** Associations between the original, 3-factor structure of the CPTI and conduct problems.

*Notes.* Each variable is depicted as a circle and lines between circles indicate unique associations (i.e., a partial correlations for which the corresponding 95% credible interval does not include zero). Blue lines indicate a positive association and red lines a negative association. Variables are placed such that strongly associated variables are placed closely
